# Emotional State of Mexican University Students in the COVID-19 Pandemic

**DOI:** 10.3390/ijerph19042155

**Published:** 2022-02-14

**Authors:** Maria Dosil-Santamaria, Naiara Ozamiz-Etxebarria, Nahia Idoiaga Mondragon, Hiram Reyes-Sosa, Javier Santabárbara

**Affiliations:** 1 Department of Research and Diagnostic Methods in Education, University of the Basque Country UPV/EHU, 48940 Leioa, Spain; maria.dosil@ehu.eus; 2 Department of Psychosocial Processes and Health, University of the Basque Country UPV/EHU, 48940 Leioa, Spain; naiara.ozamiz@ehu.eus; 3 Department of Developmental and Educational Psychology, University of the Basque Country UPV/EHU, 48940 Leioa, Spain; 4 Department of Social Psychology, Universidad Autónoma de Coahuila, Saltillo 25280, Mexico; hiram.reyes@uadec.edu.mx; 5 Centro de Investigación Biomédica en Red de Salud Mental (CIBERSAM), Ministry of Science and Innovation, 28029 Madrid, Spain; jsantabarbara@unizar.es; 6 Department of Microbiology, Pediatrics, Radiology and Public Health, University of Zaragoza, C/Domingo Miral s/n, 50009 Zaragoza, Spain; 7 Aragonese Institute of Health Sciences (IIS Aragón), 50009 Zaragoza, Spain

**Keywords:** stress, anxiety, depression, university students, chronic illness, gender, COVID-19

## Abstract

Background: Since the WHO declared the COVID-19 crisis a pandemic in March 2020, the young population is suffering from a range of psychological symptoms. The present study measured symptoms of stress, anxiety and depression in university students of Saltillo, Mexico, using the Depression and Anxiety Stress Scale-21 (DASS-21). Methods. The DASS-21 scale and an ad hoc questionnaire were used to collect sociodemographic information. Results: The results show that the students who participated in this study suffer from high levels of stress, anxiety and depression. In terms of sociodemographic variables, women, people suffering from a chronic disease and people living with a chronic disease had the highest levels of stress, anxiety and depression, and people who live with a chronically ill person, people who have had the COVID-19 disease and those who have had someone close to them fall sick have had more stress, anxiety and depression than the rest. Another finding of the present study is that university students who believe that others comply with COVID-19 safety measures have significantly lower anxiety and depression than those who believe that others do not comply. Conclusions: It is concluded that university students are a psychologically vulnerable group in the face of the pandemic.

## 1. Introduction

Nineteen months have passed since WHO declared the COVID-19 crisis as a pandemic [1]. This pandemic has brought significant change in all countries of the world. In Mexico, the first case of COVID-19 was detected on 27 February 2020, but spread exponentially from April onwards [2]. In addition, Mexico is the 23rd country in terms of the number of infections, but the third in the number of deaths in the world [3].

The impact that COVID-19 has had on the daily lives of citizens is evident [4]. Several studies have highlighted its effects on various aspects, for example, on psychological well-being [5], on economic issues, such as unemployment [6], and other studies have even come close to showing less visible effects for example, the increase in certain types of violence, such as gender-based violence [7]. The aforementioned effects allow us to position COVID-19 as a social fact because this (external) phenomenon has modified daily practices.

Therefore, the changes brought about by the pandemic have been studied worldwide at the physical, emotional, political, economic and social health levels [8]. Regarding psychological issues, hundreds of studies worldwide show that the population has suffered psychological symptomatology due to the side effects of the virus itself [9]. Many factors have been studied to see which groups have been the most vulnerable, and, in terms of age, many studies have observed that university students have suffered the most from the pandemic, having presented high levels of psychological impact [10,11].

The closure of universities [12,13], uncertainty about the future, the importance of interaction with peers and educational insecurity may have influenced the suffering of the youth. Boredom, difficulties in participating in sports activities and going out with friends could also have influenced the psychological state of young people [14], given that this is a time when the relationship with peers is very necessary for them. It cannot be forgotten that peers are the most important people during the juvenile stage, so this need to get together is understandable, since the loneliness they have suffered has led them to experience depressive symptoms [15].

Since the pandemic began, an increase in unhealthy habits among young people has been observed. Increased consumption of technologies, the use of screens, gambling, online games, substance abuse and inadequate nutrition are some of the examples [16]. In addition, being connected to the screen has also influenced the increase in stress levels [17,18]. Likewise, the distress and anxiety experienced by young people has led them to increased substance use [19] and increased emotional eating [20].

The predominant psychological symptomatology since the beginning of the pandemic have been stress, anxiety, and depression. Long periods of confinement have created much uncertainty among young people [21]. In general, this has been an ongoing situation worldwide, and it is clear that symptomatology has grown as the length of this uncertainty has increased [22]. In addition, living through the deaths caused by COVID-19 has created psychological symptoms in the general population.

People with chronic physical pathologies, as well as mental disorders, have suffered a parallel epidemic of fear, anxiety and depressive symptoms in a period in which the healthcare system has collapsed. People with chronic illness or those living with a chronically ill person have suffered the most since the beginning of the pandemic [12]. The fear of contagion together with the need to increase protective measures against it may have been the cause of this suffering.

In terms of gender, all studies intentionally conducted have shown that women have suffered the most psychological distress in the pandemic [23]. Women have suffered more because commerce, health and social services, hospitality and education are some of the sectors most affected by the pandemic, and in these sectors, the majority of workers are women [24].

As far as age is concerned, although studies conducted worldwide affirm that young people are a population whose mental health is especially affected in this pandemic, there are few specific studies on which age ranges are most affected [25,26]. However, the studies that have been found point to students between the ages of 21 and 25 or those over the age of 21 as being most affected by the pandemic [27,28].

Young people are the future of our countries, and it is essential to know how they are coping emotionally with the pandemic situation. For this reason, the present study examines the psychological well-being of young Mexican university students after one year of the pandemic. In particular, their levels of stress, anxiety and depression will be measured, as these have been the main symptoms shown by this population.

Based on this context, the main objective of this study is to analyze the levels of stress, anxiety and depression among young Mexican university students in the city of Saltillo. Concretely this research aims (1) to assess whether psychological symptoms persisted after one and a half years of the COVID-19 pandemic in a Mexican sample of university students; specifically, we aimed to determine whether there were significant differences between age groups (under 21 and over 21) and gender, and (2) to analyze whether pandemic-specific variables (having a chronic illness, living with a person who has a chronic illness, having contact with a COVID-19 positive person, having been infected with COVID-19, having a close person who has died or believing that people are respecting the measures imposed) are related to psychological symptomatology.

## 2. Materials and Methods

### 2.1. Participants

This study recruited a total sample of 252 university students aged 18–29 years (M = 21.12; SD = 3.21) from the city of Saltillo, Mexico. Of the participants, 34.1% (n = 86) were male and 65.9% (n = 166) were female.

### 2.2. Measures and Instruments

An ad hoc questionnaire was used to collect sociodemographic information related to chronic disease, living with a chronically ill person, having been infected by COVID-19, having a close person or family member who has died because of COVID-19 and their perception of whether or not people respect health measures (Likert scale).

Symptoms of anxiety, depression and stress were measured using the Depression and Anxiety Stress Scale-21 (DASS-21) [29].The DASS-21 scale is composed of 21 items and respondents were asked to report on experiences during the previous week. Responses are scored on a Likert-type scale with scores ranging from (0 = Did not apply to me) to (3 = Applied to me very much or most of the time). The three subscales of the DASS 21 have seven items for depression, seven for anxiety and seven for stress. Each subscale is scored from 0 to 21. To categorize symptoms of stress, anxiety and depression, the cut-off points discussed by Antony et al. [30] are used: no symptoms, mild, moderate, severe, severe and extremely severe. The DASS-21 has acceptable reliability and good validity [19]. Regarding reliability in our study, the Cronbach’s alpha coefficient for the depression scale was 0.89, for the anxiety scale 0.92 and for the stress 0.90.

### 2.3. Study Design

The sample was recruited using a non-probability sampling procedure. The Google Forms questionnaire that was created was sent to university students thought social networks of the university. The questionnaire explained the objectives of the study and the procedures to be followed during the study. It also indicated that they had the right to voluntarily withdraw from the study if necessary. The questionnaires informed participants of the voluntary nature of their participation and the commitment required to start the test. The Ethics Committee of the University of the Basque Country (UPV/EHU) (code M10/2020/070/R1) approved the study. All the canons established by Organic Law 15/99 on Personal Data Protection were followed for data collection. Therefore, the procedures followed were in accordance with the Declaration of Helsinki of the World Medical Association.

### 2.4. Data Analysis

Data analyses for this study were conducted using the SPSS v.26 statistical package (SPSS, Inc., Chicago, IL, USA), several of the data were categorized for further analysis. The two assumptions of normality and homogeneity of variances were confirmed. Homogeneity of variances were confirmed prior to the corresponding analysis to decide whether to use parametric or nonparametric tests. Specifically, the critical level of *p* < 0.05 of the Kolmogorov–Smirnov statistic was established and analyzed to determine the distribution of the data for the analysis of group differences.

It should be noted that the categories of depression, anxiety and stress were classified using the cut-off scores of the instrument to obtain the different levels (mild, moderate, severe and extremely severe). Likewise, a dichotomous recategorization of the Likert-type variable was performed to meet the norms.

Descriptive analyses of the independent variables and independent samples mean comparison analyses were performed using Student’s t-test. For the calculation of the magnitude of the results, the effect size was interpreted by Cohen [31] (1988).

## 3. Results

### 3.1. Descriptive Analysis of the Independent Variables of the Sample

Of the total sample, 8.7% (n = 22) reported having a chronic disease versus 91.3% (n = 230). Of the total sample, 25.4% (n = 64) reported living with a person with a chronic disease versus 74.6% (n = 188) who reported not living with a person with a chronic disease. However, 60.3% (n = 152) reported having been in contact with a COVID-19-infected person versus 39.7% (n = 100) who reported not having been in contact with any COVID-19-infected person. Likewise, of the total sample, 28.6% (n = 72) indicated having been infected by COVID-19 versus 71.4% (n = 180) who responded not having been infected so far by COVID-19. In the question related to the loss of family members to COVID-19, 37.3% (n = 94) responded that they had lost a family member to COVID-19 during this time and 62.7% (n = 158) had no family member who had died from COVID-19. Regarding the perception of compliance with the measures that have been imposed, 10.3% (n = 26) indicated that they are not being complied with at all, 81% (n = 204) very little, 7.9% (n = 20) quite a lot and 0.8% (n = 2) a lot (See Table 1).

### 3.2. Frequencies and Percentages of University Students with Different Symptomatologies According to Sociopersonal Variables

Table 2 and Table 3 below shows the frequencies and percentages of the different levels (mild, medium, severe and extremely severe) of the symptomatology under study (depression, anxiety and stress) of the participants, university students aged between 18 and 30 years, according to the independent variables analyzed (suffering from chronic disease, living with a chronically ill person, having been in contact with COVID-19-infected persons, having been infected with COVID-19, someone close to them having died of COVID-19 and respect for the measures imposed to deal with the current health crisis). However, it is important to note that out of these categories, out of the total sample, 91 people showed no level of depression, 109 no level of anxiety and, finally, 106 people no level of stress.

### 3.3. Analysis of Significant Differences between Symptomatology and Sex and Age

Table 3 shows the descriptive results and the statistically significant differences according to sex and age. The only symptomatology showing statistically significant differences are depression [t (474) = 2.84, *p* > 0.005, dcohen = 0.34], and anxiety [t (474) = 2.71, *p* > 0.007, dcohen = 0.32], only with sex, both with a moderate effect size. None of them show significant differences with age.

Table 4 below shows the statistical differences according to the symptomatology (depression, anxiety and stress) and sex, with a moderate effect size, with women scoring higher than men in the sample. In contrast, the age ranges do not show statistically significant differences with the symptomatology.

Table 5 below shows the statistically significant differences between the symptomatology and the independent variables in this study. It can be seen that the vast majority of the variables are statistically significant, with high moderate effect sizes.

## 4. Discussion

The main objective of this study was to analyze the levels of stress, anxiety and depression among university students in the city of Saltillo, Mexico a year and a half after the start of the COVID-19 pandemic. To our knowledge, this is the first research conducted in this region at this time (as almost all research was conducted in the early phase of the pandemic). Therefore, it provides valuable information to understand how a global pandemic that has lasted more than 18 months is affecting the mental health of young people and how different sociodemographic and pandemic-related variables may vary this symptomatology.

The descriptive results of the present sample represent a sample of healthy young people, since most of the participants (91.3%) do not have any chronic disease. Chronic diseases are more frequent among the older population [32]; therefore, there is not a high prevalence of chronic diseases in the present sample. In the present study, more than half of the university students (60.3%) had been in contact with a person infected by COVID-19 and furthermore, 28.6% indicated having been infected by COVID-19. This may be because at the time of the study, the pandemic had been going on for a year and a half and more and more people were infected or because young people have been infected more than older people [33], so that many participants have been in contact with COVID-19-infected person.

The proportion of young people who have lost someone close to them to COVID-19 (37.3%) is relatively high, so it could be said that there is a high proportion of university students who have lost a close person in the present sample. This is because Mexico is one of the countries with the highest mortality due to COVID-19 in the world [34] and in addition, the age of deaths and infections due to COVID-19 is in the range of 30 and 50 years (65.85%) [34]. In addition, it is noteworthy that 81% of the sample perceives that the norms to prevent the spread of the virus are not being complied with.

The study has shown that the student population may be suffering considerably after a year and a half of the pandemic. In fact, the scores obtained in this research and the proportions of young people with anxiety, depression or stress are higher than those found in the validation of the scale in a nearby area, although it is true that no meta-analysis has been found on Mexican young people with which to compare the data [35].

Anxiety may be due to the fact that the state of alarm has been prolonged for a year and a half, something that has not ceased, and fear and suffering increase with the passage of time. The lack of control in the current situation, and the uncertainty sustained over time, has been able to provoke stress symptoms among the university student population [36]. Finally, depression may be due to generalized hopelessness and sadness in the face of the prolongation and failure to see the end of the pandemic. This depressive symptomatology is of concern, as in some cases studied, depressive symptomatology has even led to suicide [21].

As in the case of most research conducted worldwide, women have suffered the most emotional harm during this pandemic [23]. Indeed, in the present study, women reported suffering far more symptoms of stress, anxiety and depression than men. In particular, young women may have also suffered more than men because the sectors in which they work have been more affected, and they may have found it more difficult to separate personal and work life [37]. In addition, women students have had to care for family members more during the pandemic than younger men [38]. Nevertheless, it has to be also taken into account that a huge body of literature suggest that gender differences on such symptoms are common even in non-pandemic times, even though the pandemic has worsened this situation [39]. With respect to age, contrary to previous research, no differences were found between university students under and over 21 years of age [27].

As expected, young people suffering from a chronic disease have had more symptomatology than people without a chronic disease since people with chronic diseases are more vulnerable to complications from COVID-19. On the other hand, people living with a chronically ill person had the highest levels of stress, anxiety and depression [40]. This could demonstrate that Mexican university students have a great fear of infecting their family members, as shown by several studies with college students [41]. This finding shows that young people feel empathy for others, something that has been denied by criminalizing them in the media. In addition, in Mexico the population has a high comorbidity [42] having several diseases, so the Mexican population is more vulnerable at the time of being infected by COVID-19 and this can raise the levels of psychological symptomatology.

An interesting finding of the present study is that both people who have had the COVID-19 disease and those who have had someone close to them fall sick have had more stress, anxiety and depression than the rest. This could mean that having experienced this disease or having had it around creates awareness of the seriousness of the disease.

Those who have had a person close to them die of COVID-19 presented more anxiety and stress than those who have not experienced such a loss. It is common for the youth to show increased emotional symptomatology due to a near death and even more so when experiencing unexpected deaths [43]. The students were not prepared to experience a near death, as death is still considered something distant, whereas they live in the moment and do not think about the future.

Another finding of the present study is that students who believe that other people comply with COVID-19 safety measures have significantly lower anxiety and depression than those who believe that people do not comply with the rules. People who do not trust others’ compliance with the rules are more alert and distrustful of others’ behavior. This indicates that, on the one hand, people who are developing more distrust of others in the current pandemic may suffer more anxiety and depression and, on the other hand, that young people are also concerned about others’ compliance with norms. This finding breaks the stigma that young people show no concern about the current pandemic [44].

Therefore, taking into account the findings of the present study, the mental health of young people in general and university students in particular, should be given importance and they should no longer be singled out as irresponsible. Thus, measures should be taken to improve mental health and prevent possible psychological illnesses among students.

First, excessive use of the Internet should be avoided and creative activities such as music [45], painting [46], dancing [47] and writing [48] should be encouraged to counteract risky behaviors that can create psychological symptoms among university students [49]. In addition, young people should be encouraged to maintain supportive relationships with their friends, as peer relationships are essential for young people [50]. In universities and other educational and youth centers, information on managing emotions and symptoms such as stress, anxiety or depression should be provided [51]. In the world of work, young people should be given facilities to re-enter the labor market.

Finally, this study has some limitations. Firstly, one of the major limitations of this study is that it cannot be directly compared with a previous study of the initial phase of the pandemic since no meta-analysis of the region or any research carried out on the same scale has been found. This makes it more difficult to contextualize the results and will be taken into account in future research; thus, it is impossible to determinate whether the pandemic caused the observed symptomatology. Finally, it is difficult to determine how many people the questionnaire reached as it was disseminated through the university’s social networks. Therefore, we have not been able to determine a concrete response rate.

However, the strength of the present study is that, to our knowledge, it is the first study conducted after a pandemic year among the students of the city of Saltillo, and the study brings new information to the scientific community about the health status of young people in this area. Secondly, a non-probabilistic sample was used, so the generalizability of the results is limited. In particular, there may be some selection bias, as participation was voluntary; therefore, only those who were particularly emotionally affected were able to participate.

## 5. Conclusions

The present study highlights the importance of addressing the mental health of university students, and further research and solutions are needed to ensure that future adults emerge from the COVID-19 pandemic in a psychologically sound state. As far as mental health professionals are concerned, interventions for the treatment of mental disorders resulting from the pandemic should be put in place, and special attention should be paid to the most vulnerable groups among young people, which are women and those who have lost someone close to them to COVID-19.

## Figures and Tables

**Table 1:** Sample independent variables.

*Variable*	Chronic Disease (%) n		Living with a Chronically Ill Person (%) n		Contact with People Who Have Been Infected by COVID-19 (%) n			
	Yes	No	Yes	No	Yes		No	
	8.7% (n = 22)	91.3% (n = 230)	25.4% (n = 64)	74.6% (n = 188)	60.3% (n = 152)		39.7% (n = 100)	
	***COVID-19 infection (%)* n**		***Close person deceased by COVID-19 (%)* n**		***Respect for the rules (%)* n**			
					*Are not fulfilled at all*	*Very little*	*Quite*	*Much*
	28.6% (n = 72)	71.4% (n = 180)	37.3% (n = 94)	62.7% (n = 158)	10.3% (n = 26)	81% (n = 204)	7.9% (n = 20)	0.8% (n = 2)

**Table 2:** Frequencies and percentages of university students with different symptomatology (mild, moderate, severe and extremely severe) according to socio-personal variables.

		*Chronic Disease (%)* n		*Living with a Chronically Ill Person (%)* n		*Contact with People Who Have Been Infected by COVID-19 (%)* n	
		Yes	No	Yes	No	Yes	No
Depression	Mild	4.5% (n = 1)	16.1% (n = 37)	12.5% (n = 8)	16% (n =30)	13.2% (n = 20)	18% (n = 18)
	Moderate	18.2% (n = 4)	17.8% (n =41)	12.5% (n =8)	19.7% (n = 37)	18.4% (n = 28)	17% (n = 17)
	Severe	9.1% (n = 2)	9.1% (n = 21)	10.9% (n = 7)	8.5% (n = 16)	12.5% (n =19)	4% (n = 4)
	Extremely severe	45.5% (n = 10)	19.6% (n = 45)	32.8% (n = 21)	18.1% (n = 34)	27% (n = 41)	14% (n = 14)
Anxiety	Mild	4.5% (n =1)	3.5% (n = 8)	4.7% (n = 3)	3.2% (n = 6)	4.6% (n = 7)	2% (n = 2)
	Moderate	18.2% (n = 4)	17% (n = 39)	15.6% (n = 10)	17.6% (n = 33)	18.4% (n = 28)	15% (n = 15)
	Severe	0% (n = 0)	16.1% (n = 37)	14.1% (n = 9)	14.9% (n = 28)	17.1% (n = 26)	11% (n = 11)
	Extremely severe	54.5% (n = 12)	18.3% (n = 42)	32.8% (n = 21)	17.6% (n = 33)	26.3% (n = 40)	14% (n = 14)
Stress	Mild	4.5% (n = 1)	14.3% (n = 33)	7.8% (n = 5)	15.4% (n = 29)	13.8% (n = 21)	13% (n = 13)
	Moderate	4.5% (n = 1)	20% (n = 46)	18.8% (n = 12)	18.6% (n = 35)	23% (n = 35)	12% (n = 12)
	Severe	27.3% (n = 6)	14.8% (n = 34)	21.9% (n = 14)	13.8% (n = 26)	17.8% (n = 27)	13% (n = 13)
	Extremely severe	27.3% (n =6)	8.3% (n = 19)	12.5% (n = 8)	9% (n = 17)	11.8% (n = 18)	7% (n = 7)

**Table 3:** Frequencies and percentages of university students with different symptomatology (mild, moderate, severe and extremely severe) according to socio-personal variables.

		COVID-19 Infection (%) n		Close Person Deceased by COVID-19 (%) n		Respect for the Rules (%) n	
		Yes	No	Yes	No	Yes	No
Depression	Mild	8.3% (n = 6)	10.9% (n = 32)	12.8% (n =12)	16.5% (n =26)	13% (n =30)	36.4% (n = 8)
	Moderate	18.1% (n = 13)	17.8% (n =32)	19.1% (n = 18)	17.1% (n = 27)	18.7% (n =43)	9.1% (n = 2)
	Severe	13.9% (n = 10)	7.2% (n =13)	11.7% (n = 11)	7.6% (n = 12)	9.6% (n =22)	4.5% (n = 1)
	Extremely severe	27.8% (n = 20)	19.4% (n = 35)	24.5% (n = 23)	20.3% (n = 32)	23.4% (n = 54)	4.5% (n = 1)
Anxiety	Mild	4.2% (n =3)	3.3% (n = 6)	5.3% (n = 5)	2.5% (n = 4)	3.9% (n = 9)	0% (n = 21)
	Moderate	16.7% (n = 12)	17.2% (n = 31)	17% (n = 16)	17.1% (n = 27)	17.4% (n = 40)	13.6% (n = 59)
	Severe	20.8% (n = 15)	12.2% (n = 22)	18.1% (n = 17)	12.7% (n = 20)	14.8% (n = 34)	13.6% (n = 20)
	Extremely severe	33.3% (n = 24)	16.7% (n = 30)	30.9% (n = 29)	15.8% (n =25)	23% (n = 53)	4.5% (n = 37)
Stress	Mild	5.6% (n = 4)	16.7% (n = 30)	9.6% (n = 9)	15.8% (n = 25)	22.7% (n = 5)	12.6% (n =29)
	Moderate	16.7% (n = 12)	19.4% (n = 35)	18.1% (n = 17)	19% (n = 30)	4.5% (n = 1)	20% (n = 46)
	Severe	25% (n = 18)	12.2% (n =22)	22.3% (n = 21)	12% (n = 19)	9.1% (n = 2)	16.5% (n = 38)
	Extremely severe	18.1% (n =13)	6.7% (n = 12)	13.8% (n = 13)	7.6% (n = 12)	4.5% (n = 1)	10.4% (n = 24)

**Table 4:** Significant differences in the symptomatology studied as a function of sex and age.

Depression	M	SD	n	t	*p*	d	Anxiety	M	SD	n	t	*p*	d	Stress	M	SD	N	t	*p*	d
**Sex**																				
Female	8.36	6.06	166	2.66	0.008 **	0.35		6.74	5.25	166	3.63	0.001 ***	0.48		9.69	5.38	166	3.70	0.001 ***	0.49
Male	6.32	5.59	86					4.47	4.37	86					7.10	5.19	86			
**Age**																				
18–21	7.74	5.87	171	0.697	0.486			5.83	4.83	385	−0.128	0.899			8.76	5.42	385	0.161	0.872	
22–29	7.17	6.01	78					5.92	5.30	94					8.64	5.39	94			

*** *p* < 0.001; ** *p* > 0.01; * *p* > 0.05.

**Table 5:** Statistically significant differences between symptomatology and independent variables.

Depression	M	SD	n	t	*p*	d	Anxiety	M	SD	n	t	*p*	d	Stress	M	SD	n	t	*p*	d
Chronic disease																				
Yes	11.50	6.36	22	2.98	0.001 ***	0.64	Yes	10.90	6.19	22	4.11	0.001 ***	0.88	Yes	11.32	5.85	22	2.12	0.044 *	0.45
No	7.30	5.80	230				No	5.57	4.79	230				No	8.57	5.36	230			
Living with a chronically ill person																				
Yes	9.18	6.75	64	2.38	0.018 *	0.34	Yes	7.65	6.06	64	3.13	0.002 **	0.45	Yes	9.84	5.88	64	1.67	0.079	
No	7.14	5.59	188				No	5.39	4.60	188				No	8.45	5.26	188			
Contact with COVID-19																				
Yes	8.72	6.06	152	3.55	0.001 ***	0.46	Yes	6.95	5.29	152	3.90	0.001 ***	0.50	Yes	9.78	5.22	152	3.54	0.001 ***	0.46
No	6.06	5.46	100				No	4.47	4.34	100				No	7.33	5.48	100			
Infected with COVID-19																				
Yes	9.01	6.31	71	2.17	0.025 *	0.30	Yes	7.95	5.43	72	3.81	0.001 ***	0.53	Yes	10.44	4.94	72	3.06	0.002 **	0.43
No	7.13	5.74	180				No	5.17	4.71	180				No	8.16	5.11	180			
Someone close died because of COVID-19																				
Yes	8.43	5.93	94	1.56	0.119		Yes	7.45	5.14	94	3.62	0.001 ***	0.47	Yes	9.97	5.77	94	2.56	0.011 *	0.33
No	7.21	5.95	158				No	5.08	4.84	158				No	8.12	5.14	158			
Respect of measures																				
Yes	4.95	4.46	22	−2.25	−0.025 *	−0.50	Yes	3.68	3.65	22	−2.27	−0.027 *	−0.51	Yes	6.81	4.69	22	−1.81	−0.073	
No	7.92	6.03	230				No	6.18	5.24	230				No	9.01	5.49	230			

*** *p* < 0.001; ** *p* > 0.01; * *p* > 0.05.

## Data Availability

The data that support the findings of this study are available on request from the corresponding author.
